# The paradox of the effectiveness of IRS insecticides (including DDT) and its impacts on human health - what can we fix if it isn’t broken?

**DOI:** 10.1186/1475-2875-11-S1-P14

**Published:** 2012-10-15

**Authors:** Hindrik Bouwman, Henrik Kylin, Maria (Riana) Bornman

**Affiliations:** 1 School of Environmental Sciences and Development, North-West University, Potchefstroom 2520, South Africa; 2 Department of Water and Environmental Studies, Linköping University, 58183 Linköping, Sweden; 3 Department of Urology and University of Pretoria Centre for Sustainable Malaria Control, University of Pretoria, Pretoria 0001, South Africa

## 

The effectiveness of DDT and other insecticides when properly used as indoor residual spray (IRS) to combat malaria is not in question [1]. However, the high body burden of DDT of those protected is very high [2], and the human health consequences due to IRS insecticides of those protected are of great concern [1-3]. What may be questioned though are the effectiveness, health impacts, social consequences, and sustainability of some IRS alternatives. Many promising ‘silver bullets’ (using anything but IRS) to beat malaria over the last number of decades have come and gone. Yet, the one proven method, IRS, gets less recognition or attention. IRS interrupts transmission where most infections occur - the home. It is also at home where those most likely to suffer malaria - babies, children and pregnant mothers - are to be found. The negative part of the IRS message though, remains the inevitable co-exposure of the very same susceptible groups to IRS insecticides. Protection by IRS comes at a cost, creating a paradox -protection from deadly malaria may carry a health burden due to the IRS chemicals used [1,3].

Policy formulation, negotiating fora, and the development of research priorities via consensus (some possibly burdened with other agendas) seem not to be good platforms to deal with intractable paradoxes. IRS with chemicals seems out of vogue and often relegated in favour of the enticing promises of high-tech or new methods.

IRS as a method has remained almost unchanged since de Meillon pioneered it in South Africa in 1936 [4]. Combining basic biological knowledge about reproductive behaviour of the female vector mosquito with residual toxic chemicals within and close to residential areas where most infections occur, is effective at preventing transmission, but bad at preventing chemical exposure and uptake of the chemicals by residents. We believe that a vast scope of options to improve on IRS remain to be explored that, while maintaining effective transmission prevention will also significantly reduce human exposure to IRS chemicals. Options for further exploration include *inter alia:* better application, more selective areas of indoor application, mosquito irritability and repellency, better formulations, and new chemicals [1].

Maintaining a proven top-down IRS strategy supported by an effective hospital and clinic system requires a minor inconvenience but no other behavioural changes by the inhabitants [5], ecological engineering, biological interventions or modifications, or vaccinations. The mostly non-intrusive IRS allows inhabitants and communities the freedom for social interactions and economic betterment unhindered by the inconvenience of most some other current forms of preventing malaria. For the foreseeable future, IRS with adequate supporting health infrastructure will remain a mainstay of malaria prevention, will most likely have a role in malaria elimination in any endemic area, and/or will remain the fall-back method in case of failure of alternatives. In the mean time, we can and should re-evaluate what works (IRS), and make it work better.
